# Announcement: 9th International Conference on Biological Inorganic Chemistry, ICBIC9

**DOI:** 10.1155/MBD.1997.117

**Published:** 1997

**Authors:** Larry Que

**Affiliations:** Department of Chemistry University of Minnesota 207 Pleasant St. SE Minnesota Minneapolis USA


					9TH INTERNATIONAL
CONFERENCE ON BIOLOGICAL
INORGANIC CHEMISTRY
ICBIC9
July 11-16, 1999
Minneapolis, Minnesota, USA
For further information, please contact
Larry Que
Chair of the Organizing Committee
Department of Chemistry
University of Minnesota
207 Pleasant St. SE
Minneapolis, Minnesota, USA
Fax: 612- 624-7029
e-mail" que@chem.umn.edu
or visit the website of the University of Minnesota Center for Metals in Biocatalysis
(http://bioinorg.chem.umn.edu) and click on the ICBIC9 button.
117

				

